# Agenesis of the left hemi-diaphragm, the cause of a neglected dyspnea in a 65-year-old female; case report and literature review

**DOI:** 10.1016/j.amsu.2022.104958

**Published:** 2022-11-17

**Authors:** Iraj Feizi, Ali Samady Khanghah

**Affiliations:** a Department of Thoracic Surgery, Fatemi Hospital, Ardabil University of Medical Sciences, Ardabil, Iran; b Department of Surgery, Fatemi Hospital, Ardabil University of Medical Sciences, Ardabil, Iran

**Keywords:** Diaphragm, Agenesis, Dual mesh, Adult

## Abstract

**Introduction and importance:**

In its complex way of embryonic evolvement, the diaphragmatic membrane can be involved with various disorders that may partially or entirely not develop. Agenesis of the diaphragm is the term that refers to this maldevelopment. It is the more severe form of congenital diaphragmatic hernia in which intra-abdominal viscera protrude into the thoracic cavity, causing respiratory and gastrointestinal problems. Most neonates delivered with diaphragmatic agenesis do not live more than hours to days of the severity of lung immaturity. However, less than 20 affected cases have been reported so far that survived to childhood and even their adulthood period treated surgically or conservatively. We have reported a case of neglected left hemi-diaphragmatic agenesis for more than six decades, then reviewed all adult diaphragmatic agenesis cases available in the literature for 74 years.

**Case presentation:**

A 65-year-old female complaining of worsened dyspnea during the last four months, a chronic history of short breath since her fourth decade of life, and recent surgery with the diagnosis of a diaphragmatic hernia, underwent the thoracotomy twice in which unilateral diaphragmatic hernia was diagnosed then repaired.

**Clinical discussion:**

For the recurrence of her symptoms, she underwent a second thoracotomy, in which the final diagnosis of left hemi-diaphragmatic agenesis was made. A dual mesh patch constructed the defect. The post-operation period was uneventful. We only found 17 cases of adult hemi or bilateral agenesis of the diaphragm reviewing the main medical literature such as Medline and Web of Science. The conservative and operative treatment managements were equal for eight patients in each of them. One of them refused therapy, and one was non-declared in the study. As in congenital diaphragmatic hernia, the most typical side was the left in 10 out of 18. The most complaints patients had followed by coughing and bowel obstruction was dyspnea and dyspepsia.

**Conclusion:**

Near the total of the diaphragmatic agenesis cases die in the neonatal population; remained undiagnosed or during an autopsy found. However, typically rare in the adult population, respiratory and digestive disorders are the most prevalent. It is difficult initially because diagnosing is intraoperatively, and no modality is available to help the examiner physician diagnose perinatally.

## Highlights

• Diaphragmatic agenesis is a rare anomaly seen usually in the neonatal period.
• The diagnosis is made during autopsy or surgery.
• The most common affected side is the left, and bilateral ageneses are reported only once in the literature.
• Who survive are usually not symptomatic until adulthood when bowel obstruction, pneumonia, or shortness of breath occur.
• We have only found 18 adult diaphragmatic agenesis cases for 74 years now.

## Graphical abstract

**Figure:**
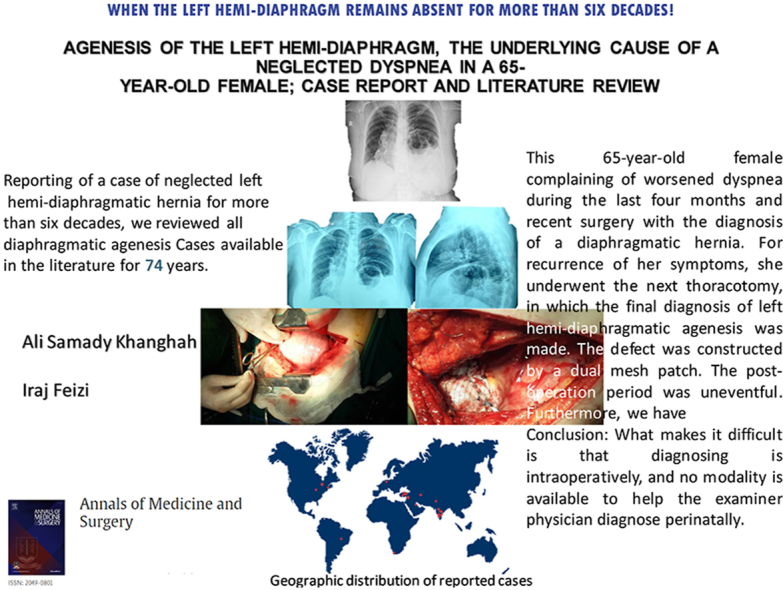

## 1 Introduction

Diaphragmatic agenesis (DA) is defined as not developing parts or the whole of the diaphragm [1]. As congenital diaphragmatic hernia (CDH), which also falls within its range, allows intra-abdominal viscera to be pushed into the thoracic cavity resulting in the death of the neonate to progressive respiratory failure with the manifestations of activity-induced and even in rest dyspnea, pulmonary hypertension [2] and gastrointestinal complaints in adults. However, these anomalies’ clinical and radiological features are similar. Regardless of being rare, most cases reported so far were newborns and often accompanied by anomalies in the other organs. Diagnosing agenesis of a diaphragm in adulthood has already been reported in less than 20 cases in the literature. The first report was about a 36-year-old female initiating her symptoms when she was three years old and constantly complaining of shortness of breath in 1948. The author insisted on the pulmonary agenesis and the absence of an ipsilateral hemidiaphragm [3]. Having reported a case of left hemi-diaphragmatic agenesis diagnosed intraoperatively in the educational and therapeutic Fatemi Hospital, Ardabil, Iran in a 65-year-old female with a history of tolerable activity dyspnea for more than thirty years that was exacerbated in this age, we reviewed the adult cases reported since 1948 in the literature. For this, we searched PubMed, Scopus, Web of Science, and Google scholar databases for 74 years ago. This work has been reported in line with the SCARE criteria [4] and also been in the list of Research Registry with the Unique Identifying Number of researchregistry8319”.https://www.researchregistry.com/browse-the-registry#home/registrationdetails/6324d4e828d6cb00212005f4/

## 2 Presentation of case

**Fig. 1:**
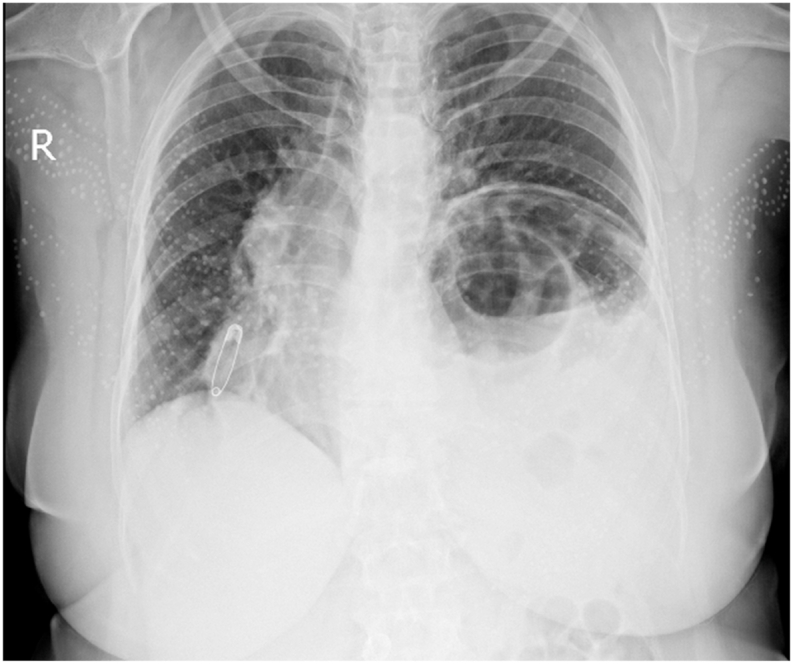
Plain PA chest x-ray in the first investigation side showing the entrance of splenic flexure of the colon into the left thoracic cavity besides mediastinal shifting and right inferior collapse of the right lung.

The patient was an overweight 65-year-old female from a low socioeconomic status who complained of exacerbated dyspnea during the last four months and was presented to the thoracic surgery centre. Having no pieces of evidence of chronic obstructive pulmonary, any proven cardiopulmonary diseases, or bowel obstruction history but only occasional constipation; however, she has been suffering from activity-related dyspnea since her third decade of life. She took only herbal laxatives and did not have trauma or prior chest surgery. The familial history from the point of any cardiothoracic disorders was unremarkable too. The grade of the chronic difficulty in breathing according to the modified medical research council (mMRC) dyspnea scale was +1. However, she was coerced into referring to a pulmonologist as the scale advanced to +3 in this period. During a physical examination, although not cyanotic, she complained incessantly of being suffocated. The oxygen saturation level was in a normal range. Notably, besides tachypneic clear respiratory sounds at the right hemithorax, bowel sounds were heard from the base to the half of the left hemithorax with decreased left-sided air entry. Blood gas analysis showed only mild respiratory acidosis with some degrees of metabolic compensation. The routine haematological and biochemical analysis gave normal results. Upright chest radiography revealed gaseous shadows suggestive of gastrointestinal components' entrance into the left hemithorax compatible with the left diaphragmatic hernia at first sight [Fig. 1].

**Fig. 2:**
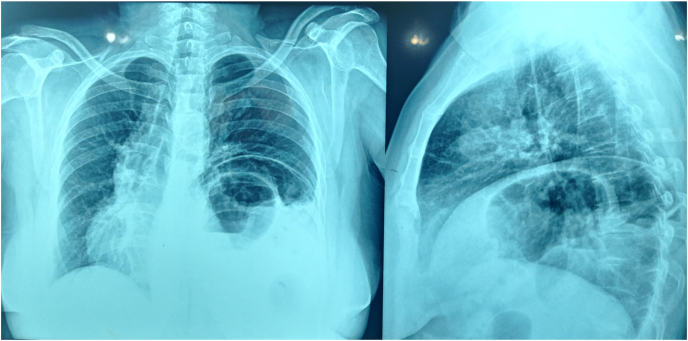
Plain PA and lateral x-ray in the second referring of the patient confirming the relapse of the event.

**Fig. 3:**
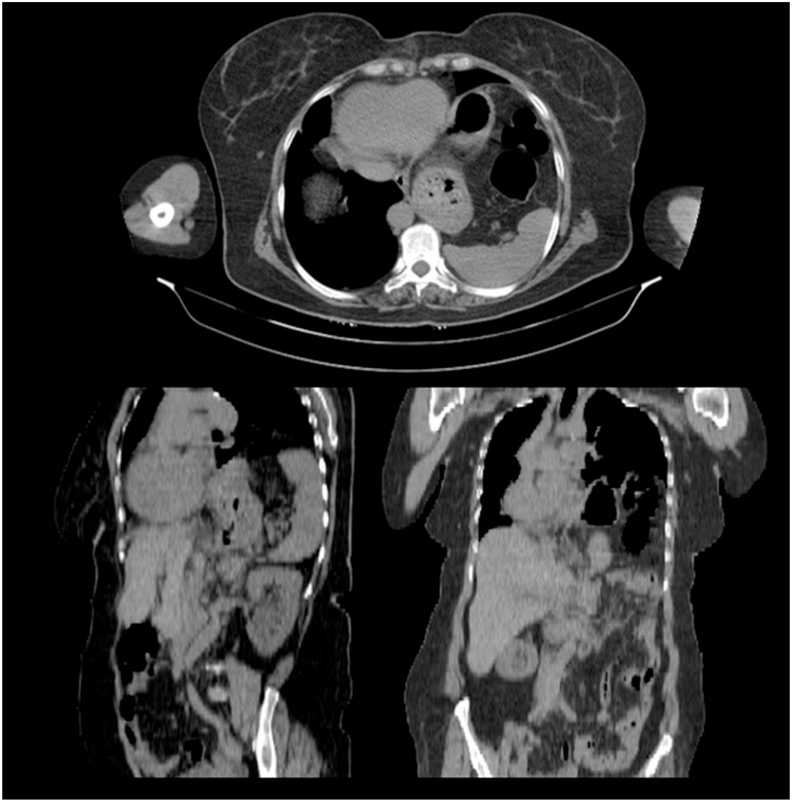
Axial CT scan at the above corroborating the x-ray founds. Accordingly, there are sagittal and coronal reconstructions at the lower left and the lower right of the picture.

**Fig. 4:**
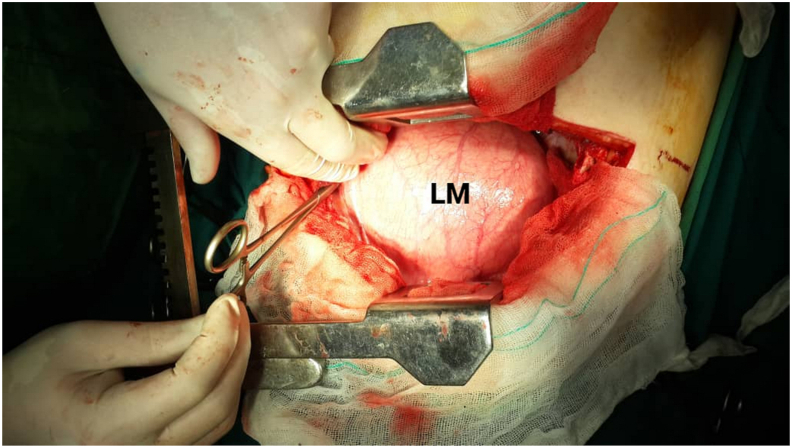
The surgical team confronted a loose membrane (LM) instead of the normal left hemidiaphragm.

**Fig. 5:**
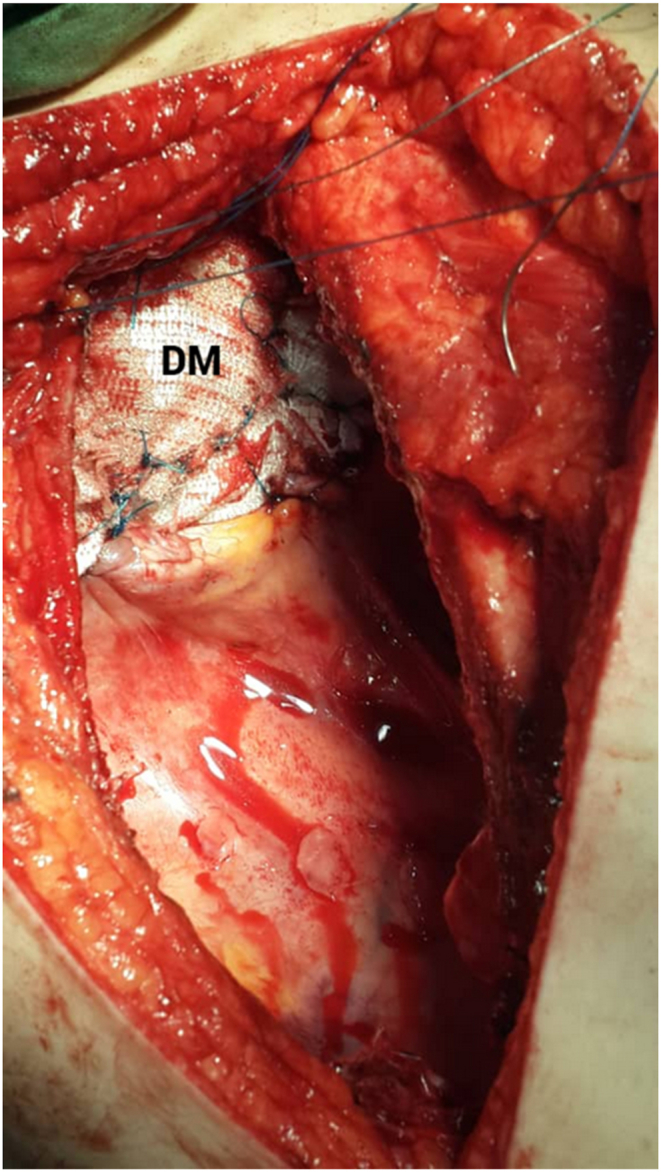
Rebuilding of the hemidiaphragm with the dual mesh (DM).

**Fig. 6:**
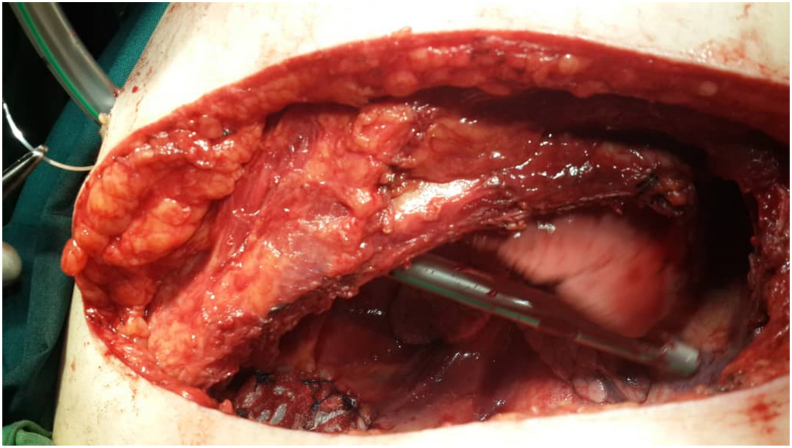
Chest tube insertion at the end of the surgery.

Thus, the patient with the preoperative diagnosis of left unilateral diaphragmatic herniation underwent surgery by a general surgeon. The complaint disappeared for about four months while the initial symptoms reoccurred. Renewed evaluations such as chest X-rays were representative of recurrent hernias at first sight [Fig. 2]. An axial chest CT scan without contrast was performed in which the absence of a diaphragmatic remnant was proved [Fig. 3]. In the second operation by the thoracic surgeon after the left lateral thoracotomy in the seventh intercostal space and getting into the hemi-thoracic cavity, the surgical team encountered vestiges of a very thin and loose membrane separating thoracic and abdominal cavities [Fig. 4]. Most of the stomach and spleen, splenic flexure, and the length of the jejunum were found in the left thoracic cavity beside a hypoplastic lung. With the final diagnosis of diaphragmatic agenesis, the existing defect was repaired by constructing a new diaphragm using a dual mesh synthetic patch and fixed anterolaterally, posteriorly, and medially with mediastinal fascia [Fig. 5]. After fixating the mesh, the left lung was inflated with high positive pressure, and a chest drain tube was inserted [Fig. 6]. The control x-ray confirmed the neodiaphragm's proper position without intrathoracic herniation of digestive system organs.

Extubating in less than 24 hours, her respiratory discomfort improved. The postoperative recovery and follow-up durations have been uneventful for two years now.

## 3 Discussion

In its complex way of embryonic development, the diaphragm, the main fibromuscular contractible organ of ventilation, can be involved with various disorders in which the respiratory and digestive systems are also affected. The four major embryologic fountains—the septum transversum, pleuroperitoneal membrane, dorsal oesophagal mesentery, and the body wall-join in developing this dome-shaped septum that separates the abdominal and thoracic cavities around the third-fourth month of gestation [5]. During the third week of gestation, the fusion of the transverse septum with the dorsal mesentery of the foregut creates two openings whereby the thoracic and abdominal contents meet [6]. Left diaphragmatic defects are more common than the right side, probably because of an earlier closure of the right pleura-peritoneal hiatus [7]. Congenital diaphragmatic maldevelopment spectrum, whether hernias, eventration, or agenesis, can lead to lung hypoplasia, pulmonary hypertension, and also digestive tract obstruction symptoms [8]. However, for the extent of the defect, most of the cases get symptomatic in early childhood. Lung hypoplasia and immaturity occur in cases of the ipsilateral side of the defect. The two hypotheses explaining this fact are the maldevelopment during the stages of organogenesis resulting in bilateral hypoplasia, followed by compression of the ipsilateral lung secondary to the herniation of the abdominal viscera at later stages [9]. A reduction in the total pulmonary vascular bed for decreased number of vessels per unit of the lung, along with pulmonary vascular remodelling, is responsible for pulmonary hypertension in congenital diaphragmatic agenesis or hernias. Pulmonary hypertension, apart from any aetiology, can lead to right ventricle (RV) dysfunction. Cervical somites are the sources of muscle precursors of the diaphragm. Migrating to the pleuroperitoneal folds, they start to proliferate and differentiate. The genes responsible for this process interact with one another in the embryonal pathway for the development of the brain, heart, lungs, diaphragm, kidneys, and pancreas [10]. Therefore, multiple anomalies are present in patients with CDH and DA.

CDH is the most well-known developmental diaphragmatic disorder due to a failure of the embryonal posterolateral pleuro-peritoneal folds to migrate anteriorly, separating the thoracic and abdominal cavities. Its prevalence varies and is estimated at less than three per 10000 births, according to the reports [11]. CDH has overall high infant mortality rates, in case only two-thirds of affected live-born infants survive to their first birthday [8]. However, the agenesis of the diaphragm is defined as an absence of a part, a hemidiaphragm, or the whole of it. Simply the vast diameters of defect, the higher mortality in the initial months of life.

DA was initially described by Gregers Thomsen as a primordial defect in the development of the lung, corresponding to similar defects in other organs in a thirty-six-year-old woman complaining of shortness of breath and cyanosis after physical activities [3]. We have reported a case of neglected hemi-diaphragmatic agenesis for more than six decades. Then review the previously reported cases of diaphragmatic ageneses, whether uni or bilateral, since 1948 as far as possible. Table 1 has ordered the 17 cases by the year reported and describes in detail of author, initial presentation(s), age and sex, site of involvement, co-existing problems, and outcomes finally [[12], [13], [14], [15], [16], [17], [18], [19], [20], [21], [22], [23], [24], [25], [26], [27]]. The databases used were PubMed/Medline, Google Scholar, and Web of Science. The inclusion criteria were only ages higher than 19 years old. Therefore neonatal and pediatric populations, diaphragmatic hernia or eventration, and cases diagnosed via autopsy were excluded from this study.

**Table 1:** Title: Previously reported cases of diaphragmatic agenesis.

No.	Authors	Year	Presentation	Age and sex	Site	Co-existing problems	Outcome
1	Gregers Thomsen	1948	Dyspnea, Cough	36-year-old female	L	Ipsilateral pulmonary agenesis	Non-declared
2	George. Tzelepis et al.	1988	Cough	22-year-old male	L	Left lower lobar infiltration in CXR	Conservative treatment
3	John J. Sheehan et al.	2000	Bowel obstruction	66-year-old female	L		Conservative treatment
4	Abel Wakai et al.	2000	Progressively Fatty food intolerance	41-year-old male	R		Conservative treatment
5	Christopher D. Anderson	2003	Nausea, vomiting, and severe epigastric pain	51-year-old female	B		PTFE mesh repair
6	Landino Fei et al.	2008	Dyspepsia	71-year-old man	L	Bowel obstruction	ePTFE patch repair
7	Hye Young Sung et al.	2009	Chest pain	70-year-old female	L	Vomiting	Conservative treatment
8	Dimitrios pousios et al.	2010	ARDS	24-year-old male	L	Vague abdominal pains	Dual mesh reconstruction
9	A. Jha	2013	Dyspnea	25-year-old female	R		ePTFE mesh repair
10	Syed Asmat Ali et al.	2013	Dyspnea	25-year-old female	R		Polypropylene mesh repair
11	Vinod Bhan et al.	2013	Pneumothorax	22-year-old male	L		Polypropylene mesh repair
12	Janilson de Sousa Cavalcante et al.	2015	Right flank pain	40-year-old female	R		Conservative treatment
13	Julide Sagiroglu et al.	2016	Progressive dyspepsia	36-year-old female	R	Cholecystitis	Cholecystectomy without further treatments
14	Binay Krishna Sarkar et al.	2016	Cough	62-year-old male	L	Recurrent lower respiratory infections, Lung cysts	Cystectomy, conservative treatment for DA
15	Aylin Okur et al.	2016	Chest pain	70-year-old female	R		The patient refused the operation
16	Sivaprabu	2017	Bowel obstruction	33-year-old male	R		Prosthetic mesh repair
17	Angela Gurradoa	2018	Bowel obstruction and dyspnea	64-years-old woman	L	Toxic megacolon	Sub-total colectomy conservative care for DA

Table abbreviations: L: left-sided, R: Right-sided, B: Bilateral, CXR: Chest x-ray, PTFE: Polytetrafluoroethylene, ePTFE: Extended PTFE.

Almost always, agenesis leads to the death of the patients in their first hour or days of life, while those who survive to adulthood are not symptomatic until their third decade [[18], [19], [20], [21],^28^]. The majority of middle-aged patients had presented with gastrointestinal problems [[13], [14], [15], [16], [17],^23^,^26^]. Otherwise, no other cardiac or asthmatic symptoms, and the ages of being symptomatic and the age of diagnoses were notable in our case. There were similarities in imaging findings and the primary diagnosis of diaphragmatic hernia. However, the main difference between ours and the first reported case was in the presence of an ipsilateral lung. Our patient has a hypoplastic left lung pushed superiorly by the stomach and bowel loops. Bowel obstruction symptoms were also absent. Pulmonary complications' appearance in the first minutes or hours after birth makes congenital diaphragmatic herniation diagnosis easier [29]. However, in the cases of partial or even complete agenesis of a hemidiaphragm, the patient has no or subtle complications unless worsening of dyspnea or bowel loops obstruction into the thorax urges them to a surgical centre. In complete Hemi-diaphragmatic agenesis, no diaphragmatic remnant is present, but in partial agenesis, a small rim of the diaphragm may be present in the posterior aspect [13]. Simply, respiratory and digestive complaints were the most commonest, including dyspnea, coughing, chest pain, and acute respiratory distress syndrome. On the other hand, dyspepsia and bowel obstruction were the most prevalent consequently in each category.

Pre-operating Diagnosis of DA is almost impossible. Thus, whether the diagnosis is intraoperatively is done mainly for bowel obstruction in diaphragmatic hernia or during autopsy. Although sporadic, genetic consultation is recommended for future pregnancies in the cases of CDH or DAs leading to neonatal death. Bilateral DA is a much rarer entity than bilateral CDH.

Answering the question of when to repair the agenesis of the diaphragm is the basis of surgically. Why so? For bowel incarceration and strangulation risk in cases of partial defects or smaller size, immediate repair is unanimously suggested, even in asymptomatic patients [30]. Of course, the patient will be lucky to be diagnosed before becoming symptomatic. On the other hand, surgery for larger asymptomatic defects remains a dilemma yet. Although primary closure is the mainstay treatment method for many diaphragmatic hernias, in the cases of larger defects or agenesis, direct suturing maybe not be practicable because of the absence of adequate muscular tissue or adhesions [16,31]. Absorbable meshes should be abstained from their lack of appropriate life-long support due to rapid absorption. Prosthetic material has been introduced as different literary techniques for creating a new diaphragm [29]. Various methods have been proposed for repairing these defects, such as direct suturing to the liver, prerenal fascia, free grafts, abdominal or intercostal muscle flaps, the use of prosthetic material, and the bovine pericardium [32]. Reconstruction was performed in this case with dual mesh. Repairing with this method has been reported rarely [26].

Cardiopulmonary function and associated anomalies are mainly the predictor factors for the affected patients' survival rate rather than the defect's size.

Delayed surgery and postmortem diagnosis made biases in both series that are divergent criteria for identifying and interpreting the results. Still, certain is that DA is more fatal than CDH.

## 4 Conclusion

In the opinion of most of the literature, DA is settled at the end of the spectrum of CDH as the most severe form of it. On the other hand, the larger the defect, the poorer the prognosis. Most neonatal cases die in the first hours and days of life because of corresponding lung hypoplasia or pulmonary hypertension. Those who reach adulthood are not severely symptomatic until adulthood when shortness of breath or even bowel obstruction persuades them to a medical centre. One-third of cases reported were in their third decade of life. All of them presented with respiratory disorders. Therefore, it could be concluded that breath-related problems outburst earlier.

Furthermore, those who had tolerated lung-related problems until their middle ages had been referred with digestive complaints of dyspepsia or even intestinal obstruction. It is difficult to diagnose DA intraoperatively, and no modality is available to help the examiner physician diagnose perinatally. Although so rare, numerous adult cases probably did not undergo surgical treatment or were misdiagnosed as diaphragmatic hernias, so the number of reports in the literature is such few that it does not reach 20.

## Ethical approval

The ethics committee of Ardabil university of medical sciences has proved and observed all aspects of this research from proposal to the final form of the manuscript. If necessary, the corresponding author can submit the certificate.

## Sources of funding

We authenticate not receive any financial support.

## Author contribution

Iraj Feizi was the surgeon who operated on the patient, suggested publishing a case report. Ali Samady Khanghah, a member of the research committee of the hospital prepared the manuscript, decided to advancing it to a “case report and literature review” manuscript, and then pursuits the submission process.

## Registration of research studies

1. Name of the registry:Agenesis of the Left Hemi-diaphragm, the Underlying Cause of a Neglected Dyspnea in a 65-Year-Old Female; Case Report and Literature Review
2. Unique identifying number or registration ID:researchregistry8319
3. Hyperlink to your specific registration (must be publicly accessible and will be checked):https://www.researchregistry.com/browse-the-registry#home/registrationdetails/6324d4e828d6cb00212005f4/

## Guarantor

Ali Samady Khanghah accepts full responsibility for the work and approves the whole process from designing the study to publish.

## Consent

The consent in which the patient has allowed to use medical records and therapeutic information is attached to the medical document. The authors testify the patient privacy maintenance. A copy of the written consent is available for review by the Editor-in-Chief of this journal on request.

## Provenance and peer review

Not commissioned, externally peer-reviewed.

## Declaration of competing interest

The authors declare that there are no conflicts of interest preparing this manuscript and accept any responsibilities.
